# Prefiltering based on experimental paradigm for analysis of fMRI complex brain networks

**DOI:** 10.1371/journal.pone.0238994

**Published:** 2020-10-14

**Authors:** Salvador Jiménez, Laura Rotger, Carlos Aguirre, Alberto Muñoz, Sergio Granados, Jesús Tornero

**Affiliations:** 1 Dept. Matemática Aplicada a las TIC, ETSI Telecomunicación, Universidad Politécnica de Madrid, Madrid, Spain; 2 GNB, EPS, Universidad Autónoma de Madrid, Madrid, Spain; 3 Departamento de Radiología, Universidad Complutense de Madrid, Madrid, Spain; 4 Unidad de Diagnóstico por Imagen, Hospital Los Madroños, Madrid, Spain; Unviersity of Burgundy, FRANCE

## Abstract

Brain networks offers a new insight about connections between function and anatomical regions of human brain. We present results from brain networks built from functional magnetic resonance images during finger tapping paradigm. Pearson voxel-voxel correlation in time and frequency domains were performed for all subjects. Besides this standard framework we have implemented a new approach consisting in filtering the data with respect to the fMRI paradigm (finger tapping) in order to obtain a better understanding of the network involved in the execution of the task. The main topological graph measures have been compared in both cases: voxel-voxel correlation and voxel-paradigm filtering plus voxel-voxel correlation. With the standard voxel-voxel correlation a clearly free-scale network was obtained. On the other hand, when we prefiltered the paradigm we obtained two different kind of networks: 1) free-scale; 2) random-like. To our best knowledge, this behaviour is reported here for first time for brain networks. We suggest that paradigm signal prefiltering can provide more infomation about the brain networks.

## Introduction

It is known, since the nineteenth century, that the brain constitutes a huge and complicated structural network [1]. The latest advances in the study of complex systems have motivated new approaches and interpretations applied to brain structural and functional characterization [2, 3].

Functional brain networks can be studied with fMRI [4]. One of the first studies where functional magnetic resonance imaging was used to extract functional networks connecting correlated human structural images of brain was performed by Eguíluz et al. [5]. In this work they reconstructed correlation matrices of BOLD signals from all MRI voxels during different finger tapping tasks for seven healthy subjects. The resulting functional networks showed small-world behaviour [6] with large clustering coefficients, from 0.14 to 0.15 for a threshold ranging from *r*_*c*_ = 0.6 up to 0.8, a short path length, and a probability of a functional connection between any two nodes (degree distribution), scaled as a power law *P*(*k*) ∝ *k*^−*γ*^ [7]. Their computations showed clearly scale-free degree distributions with a scaling exponent *γ* close to 2, a value that was identified as independent of the threshold value *r*_*c*_. A similar dependence of the functional connectivity was found by Salvador et al. [8]. Resting-state functional connectivity in the human brain was studied by van den Heuvel et al. where both patterns, the small-world configuration as well as the power law degree distribution with a scaling exponent around 2, were confirmed [9], regardless of the threshold. In all mentioned works the authors identified clearly scale-free degree distributions and compared them to random-like distributions with a marked different behaviour.

**Fig 1:**
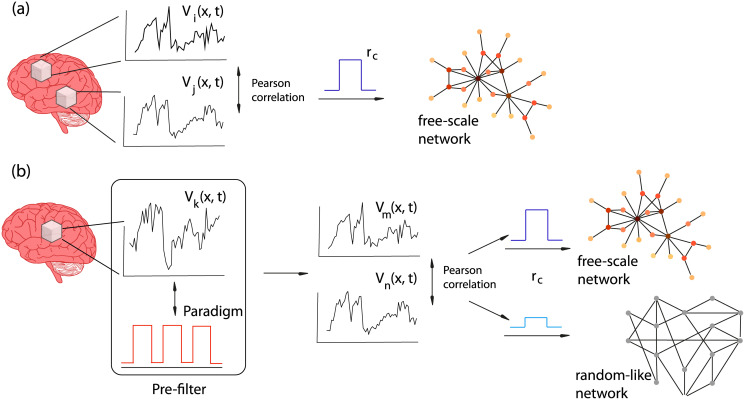
Methodology. (a) standard voxel-voxel correlation leading to a free-scale network. (b) By previous filtering with the paradigm different typologies of networks are obtained (free-scale or random-like) as a function of the threshold r_*c*_.

Following the approach of Eguíluz et al., in this work we have studied voxel-voxel correlation matrices of BOLD signals which were extracted from fMRI studies and we have constructed and analyzed the corresponding graphs. Besides this standard framework, we have implemented a new approach consisting in a previous filtering of the data with respect to the fMRI paradigm in order to obtain a better understanding of the network involved in the execution of the task. The main topological graph measures have been compared in the two cases: voxel-voxel correlation and voxel-paradigm filtering plus voxel-voxel correlation. We illustrate the process in Fig 1.

The pre-filtering is justified since it is aimed at selecting the nodes related to the task in order to enhance the effect under study. We understand that no meaningful information related to the task is removed since pre-filtering is based on correlations with the paradigm. Higher order structural properties may be eliminated by applying pre-filtering, in fact if these higher order structural properties are not related to the task we understand that they should be removed.

## Materials and methods

### MRI protocol

Five volunteers all right-handed, 4 males, 1 female, mean age 38, were recruited at Hospital Los Madroños in Madrid, Spain. A previous questionnaire, a general medical and neurological examination were performed for volunteer selection. Those volunteers were taken from a previous unpublished study (Tractografía Funcional de Imagen de Resonancia Magnética) whose protocol was previously approved from the Local Ethics Committee. As the current individuals have been selected from the former study, Los Madroños Board of Director waived any new requirement for the current study. The study was performed using a Siemens-Avanto 1.5 Tesla imaging system with a 12-element head matrix coil. The finger-tapping test (FTT) is a neuropsychological test that examines motor functioning, specifically, motor speed and lateralized coordination. Subjects, while they were comfortably lying down on the scanner stretcher, were asked to perform FTT with their index finger by flexing-extending the metacarpo-phalangeal joint. During the procedure, the subject’s palm was immobile and flat on the board, with fingers extended, and the index finder placed on the counting device. One hand at a time, subjects tap their index finger on the lever as quickly as possible within a 10 s time interval, in order to increase the number on the counting device with each tap.

Onset and end of a task were indicated by a brief light signal (≈ 1 s) generated by light-emitting diodes. For head fixation, a vacuum head holder was used. Subjects had ear plugs and were advised to keep their eyes closed during the whole examination. Task execution was controlled by on-line video monitoring.

fMRI Data Processing Gradient-echo EPI acquired during fMRI examinations were exported to a workstation with Brain Voyager software [10] for fMRI data processing. Prior to statistical analyses, the time series of the functional images were aligned for each slice to minimize the effects of head motion and linear trends. Data analyses for all studies included spatial and temporal Gaussian smoothing, removal of linear trend and nearest-neighbour cluster analysis. Correlation maps were computed with the stimulation protocol as reference function reflecting the temporal sequence of stimulation and control condition. On a pixel by pixel basis, the signal time course was cross-correlated with the reference function. Significant activation was reported at P < 0.001. We registered the mean increase of the perfusion in each region of interest (ROI). We used a minimal cluster size of six pixels considering that the subcortical ROI were smaller than the cortical motor areas.

Cubic spline interpolation was used for slice scan time correction. Images from the first dynamics were used as a reference, and translation and rotation of images in subsequent dynamics were plotted in all directions to illustrate motion [11]. Trilinear estimation and interpolation were used for 3D-motion correction. An 8-mm full width at half maximum Gaussian filter was used for spatial smoothing. Linear trend removal and a high-pass filter with 3 cycles/points were used for temporal filtering. The T1-weighted 3D images were also exported to BrainVoyager to create an anatomic image series, and the processed fMRI was coregistered to the anatomic images automatically. The 3D dataset with anatomic images and fMRI information was then transformed to the Talairach atlas to create a 3D-aligned time course dataset. A stimulation protocol was then created in BrainVoyager to represent the block design (with hemodynamic response function -HRF- refinement) used in the fMRI scans. General linear model analysis was performed to calculate activation maps for the 3D-aligned time course dataset for each subject [12]. Finger tapping paradigm was design as a sequence of activation-deactivation cycles starting with an activation state [13]. Blood oxygenation level-dependent single-shot T2* with TR 3000ms, TE 150ms, Slice thickness 8, and 64x64, 20 planes were acquired. Sagittal 3d T1 MPRAGE isotropic 256x256x1mm FOV 25.6 sequence was acquired as an anatomical basis for subsequent fusion with BOLD results. Each paradigm-cycle had a duration of 15 seconds and the finger tapping task lasted a total of 195 seconds. Thus the temporal signal for each voxel *V*(*x*_*i*_, *t*) consisted of 60 points with a sampling rate for the BOLD signal of 0.3077 Hz.

### Graph implementation

Graph theory is the mathematical study of networks. A graph is a mathematical representation of a network of elements connected to each other. A simple graph is formed by a set of nodes, or vertices, and a set of edges, or links, connecting pairs of nodes. The total number of nodes is called the order of the graph, the total number of edges is called the size of the graph. The nodes represent the elements of the system. In our case they are the voxels of a functional magnetic resonance imaging (fMRI) [2]. The links represent the connections between each pair of voxels. In our case, they mark the existence of a correlation between the time-signals of the two voxels with value above a given threshold.

This correlation has been measured by the Pearson coefficient according to the expression: [5]

$$\begin{array}{c} r\left(x_{i},x_{j}\right)=\frac{\left\langle V\left(x_{i},t\right)V\left(x_{j},t\right)\right\rangle -\left\langle V\left(x_{i},t\right)\right\rangle -\left\langle V\left(x_{j},t\right)\right\rangle }{\sigma \left(V\left(x_{i}\right)\right)\sigma \left(V\left(x_{j}\right)\right)}, \end{array}$$ (1)

where *r*(*x*_*i*_, *x*_*j*_) is the correlation coefficient between voxels *i* and *j*, *V*(*x*_*i*_, *t*) is the BOLD signal of voxel *i* as a function of time *t* and 〈⋅〉 represents the average over the time values in the series, defined as

$$\begin{array}{c} \left\langle V\left(x_{i},t\right)\right\rangle =\frac{1}{N}\sum_{k=1}^{N}V\left(x_{i},t_{k}\right), \end{array}$$ (2)

*N* being the total number of values in the temporal series. The standard deviation, *σ*, is given by:

$$\begin{array}{c} \sigma^{2}\left(V\left(x_{i}\right)\right)=\left\langle V{\left(x_{i},t\right)}^{2}\right\rangle -{\left\langle V\left(x_{i},t\right)\right\rangle }^{2}. \end{array}$$ (3)

The short time span of the signal implies that no subsampling (such as windowing or analogue techniques) was relevant to the analysis. Longer signals such as those obtained in Resting State studies may demand considering the time evolution of the signal [14, 15]. Our work focus on the influence of the different threshold values on the network not the temporal dynamics as shown in other approaches [16–19]. Since the coefficient *r*(*x*_*i*_, *x*_*j*_) is symmetric, we consider undirected links: in a pair of correlated voxels there is not a source and a destination.

The links between voxels *i* and *j* are represented in a matrix with elements *r*(*x*_*i*_, *x*_*j*_). This is the adjacency matrix. In our case, when the correlation is above the given threshold, *r*_*c*_, such that *r*(*x*_*i*_, *x*_*j*_) > *r*_*c*_, we will assign the value 1, and 0 otherwise. In this way, our effective matrices will always be considered as binary ones with all the accepted links having the same weight [20].

Once the network is, thus, created, its main topological graph measures are computed, namely the degree distribution and its Shannon Entropy *S*, the clustering coefficient *C*, the characteristic path *L* and the network efficiency *E* [21, 22].

The characteristic path of a network, *L*, is the average of the minimun paths lenghts between all pairs of nodes in the graph. These are the number of links to be traveled on the shortest route from one node to the other. Although relevant, the value of *L* can be biased by a small number of nodes very remote or even disconnected, since the logical topological distance between disconnected nodes is infinite. A more reliable measure is the network efficiency, *E*, computed from the inverses of the minimum path lengths between pairs of nodes. This avoids the excessive influence of disconnected nodes, as their efficiency has null value [2]. For node *k*, we define its characteristic length as:

$$\begin{array}{c} L_{k}=\frac{1}{n-1}\sum_{i=1}^{n}d\left(k,i\right), \end{array}$$ (4)

where *n* is the number of nodes in the graph and *d*(*k*, *i*) is the length of the shortest path connecting nodes *k* and *i*. Using *L*_*k*_ we can define the (global) characteristic path of the graph as:

$$\begin{array}{c} L=\frac{1}{n}\sum_{k=1}^{n}L_{k}. \end{array}$$ (5)

Equivalently we can define the Efficiency of node *k* as

$$\begin{array}{c} E_{k}=\frac{1}{n-1}\sum_{i=1,i\neq k}^{n}\frac{1}{d(k,i)}, \end{array}$$ (6)

Using *E*_*k*_ we define the (global) efficiency *E* of the graph as:

$$\begin{array}{c} E=\frac{1}{n}\sum_{k=1}^{n}E_{k}. \end{array}$$ (7)

The topological proximity between the nodes has a positive effect on their interaction in many networks. There are several graph measures that evaluate the organization of the network in logical topological neighbourhoods, known as clusters. One of these parameters is the clustering coefficient, *C*, that measures the density of connections between the neighbours of a node. The graph clustering coefficient is computed as the average of the clustering coefficients for each node in the graph. We define the clustering coefficient for node *k* as:

$$\begin{array}{c} C_{k}=\frac{2\Gamma (k)}{n_{k}\left(n_{k}-1\right)}, \end{array}$$ (8)

where Γ(*k*) is the number of edges between the neighbours of node *k* (thus, the number of triangles that have node *k* at one of its vertices) and *n*_*k*_ is the number of nodes that are neighbours of node *k*. The clustering coefficient of a graph with *n* nodes can be defined now as:

$$\begin{array}{c} C=\frac{1}{n}\sum_{k=1}^{n}C_{k}. \end{array}$$ (9)

A significative description of a graph can be obtained considering the degree distribution. As mentioned above, in the case of scale-free networks the degree distribution follows a power law *P*(*k*) ∝ *k*^−*γ*^, where *k* represents here the degree. A simple way to illustrate this is to represent *P*(*k*) as a function of *k* in a double-logarithmic scale since it corresponds, then, to a linear dependence with (−*γ*) as linear coefficient:

$$\begin{array}{c} P\left(k\right)=ck^{-\gamma }\Leftrightarrow \log(P(k))=\log\left(c\right)-\gamma \log\left(k\right). \end{array}$$ (10)

A measure associated to the uncertainty of a probability distribution is the Shannon entropy, *S* [23]. It measures the uncertainty in the degree of a node picked randomly. A distribution with maximun Shannon entropy is a uniform distribution, in which all events occur with the same probability. A distribution with minimal Shannon’s entropy is a Dirac delta, in which the uncertainty is zero because the result of the event is always the same. We compute the Shannon’s entropy of the degree distribution with the following expression:

$$\begin{array}{c} S=-\sum_{k=1}^{n}P\left(k\right)\log(P(k)), \end{array}$$ (11)

where *P*(*k*) is the number of occurrences of the degree *k*. We may view *P*(*k*) as a probability distribution given by the number of times that a given degree appears in the graph if we divide by the total number of nodes.

Due to the huge size of the order of the resulting graphs (≈ 2 ⋅ 10^5^) and of their size (≈ 4 ⋅ 10^9^) a new C library for graph creation and computation has been built. This library supports different types of data structures (adjacency matrix, adjacency list, hash table and double linked list) for graph manipulation, selecting the optimal type of data for the measure to be computed. The library (C source code) has been designed having as main objective to obtain a fast computing time with a low memory consumption [24]. The more demanding computations were carried out on a workstation with 3.00GHz Intel Xeon E5-2687W v4 processor and 512-GB RAM.

### Voxel-paradigm filtering

To validate the voxel-paradigm filtering as a tool with a possible clinical character, our procedure was compared to standard software, namely Brain Voyager and FSL [25]. Parametric maps were generated with selected voxels with a correlation with HRF compensated paradigm *p* < 0.012 for all the subjects with Brain Voyager software. Activation regions were located on sagittal, coronal and axial planes for healthy volunteers under a right-handed finger tapping fMRI study. Pearson correlation values between the temporal BOLD signal from the 81920 voxels obtained from the scanner divided in 20 planes of 64 × 64 cells and the HRF compensated paradigm function were performed with threshold *r*_*c*_ = 0.7 for all the subjects. Similar results were obtained when correlating BOLD-voxel signal with paradigm and from Brain Voyager postprocess.

## Results and discussion

**Fig 2:**
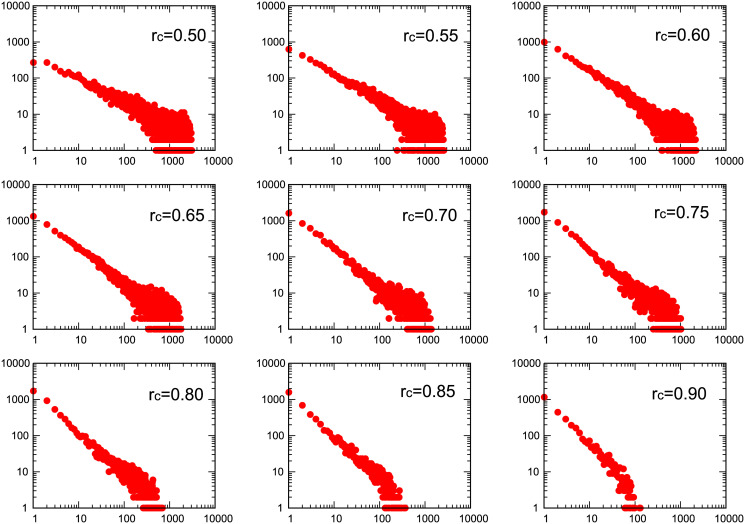
Log-log plot of nine degree distributions extracted from brain networks obtained for voxel-voxel correlation under finger tapping paradigm for subject 1 as a function of threshold value *r*_*c*_ from 0.5 up to 0.9. Horizontal axis represents the degree and vertical axis counts.

In this work, the functional brain networks are characterized through the properties of the corresponding graphs, generated for different threshold values. Brain networks are built from functional magnetic resonance images under finger tapping paradigm taken at Hospital Los Madroños, Madrid, Spain. Following the results of Eguiluz et al. [5] a Pearson correlation voxel-voxel in time domain was performed using Eq 1 for all subjects. As mentioned above, two voxels were defined as functionally connected if their temporal correlation exceeds the threshold value *r*_*c*_. Fig 2 shows log-log plot of nine degree distributions for subject 1 as a function of the threshold value *r*_*c*_ from 0.5 up to 0.9. In all plots, the horizontal axis represents the degree *k* and the vertical one axis the recounts *P*(*k*). Similar results where obtained for the rest of the subjects.

**Fig 3:**
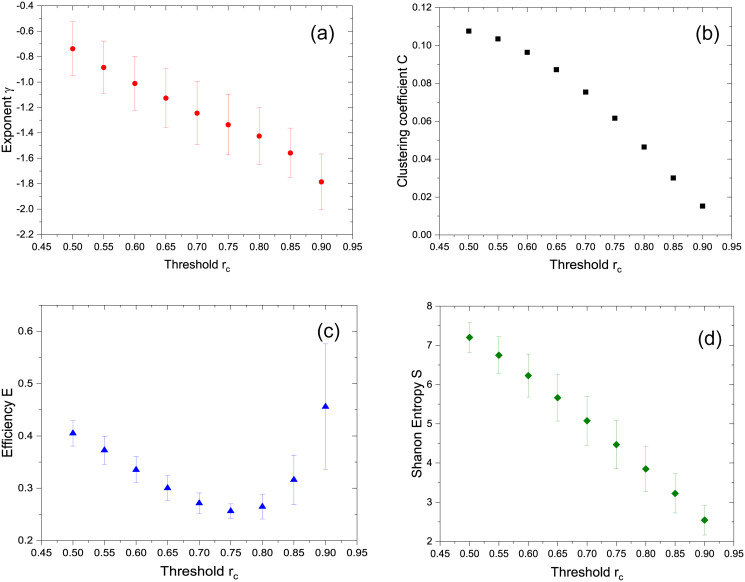
Average slope *γ* of the fittings of Fig 2 (a), clustering coefficient C for subject 1 (b) and averaged values of efficiency *E* (c) and Shannon entropy *S* (d) for all the subjects as a function of the threshold *r*_*c*_.

Log-log degree distributions for all subjects, similar to the ones in Fig 2, were fitted showing a linear behaviour. Fig 3(a) shows averaged slopes *γ* as a function of the threshold *r*_*c*_. The main topological graph measures were computed, mainly, the clustering coefficient *C*, the Shannon entropy *S*, the characteristic path *L* and the network efficiency *E* for all the subjects. Fig 3(b) shows the clustering coefficient *C* for subject 1 where rest of subjects showed similar clustering coefficient behaviour as a function of the threshold. Average values of *E* and *S* for all subjects as a function of the threshold *r*_*c*_ from 0.50 up to 0.90 are shown in Fig 3(c) and 3(d). The valur of L increases with the threshold. Contrarily to E, no relevant information seems to be obtained from L as a function of the threshold. We understand this fact is due to the presence of more disconnected nodes as *r*_*c*_ increases.

**Fig 4:**
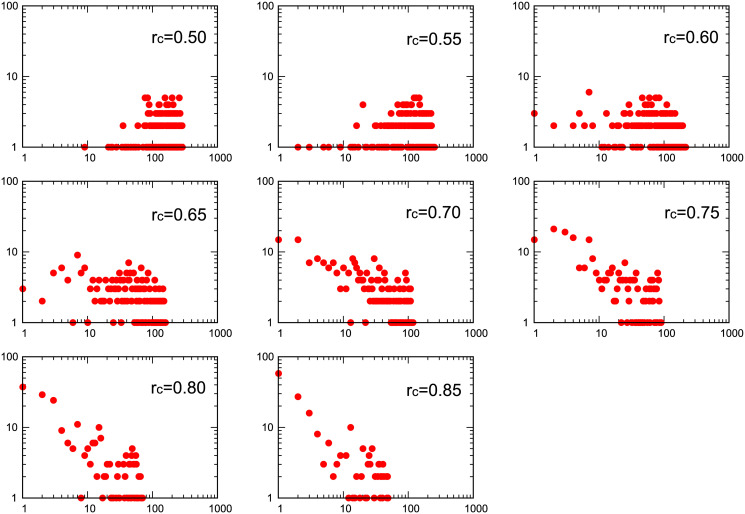
Log-log plot of eight degree distributions extracted from brain networks obtained with a previous filtering to the data correlating voxel signal with HRF compensated paradigm and a *r*_*c*_ = 0.50 threshold for subject 1. Subsequent voxel-voxel correlation was calculated for threshold values *r*_*c*_ from 0.5 up to 0.85. Horizontal axis represents the degree and vertical axis and horizontal axis counts.

As a new procedure, in order to enhance the contribution of voxels whose response might be more directly involved in the execution of the task required by the paradigm, we performed a filtering of the data taking into account the correlation of their BOLD time-signal with the one of the paradigm, convoluted with the standard HRF: only voxels with time-signal correlated to that of the paradigm with a correlation coefficient above *r*_paradigm_ = 0.5 were selected. Then, we repeated the graph construction by Pearson voxel-voxel correlations given by Eq 1. In Fig 4 we show, for subject 1 and right-handed finger tapping paradigm, eight distributions in a log-log plot as a function of the threshold value *r*_*c*_, from 0.5 up to 0.8. As before, the vertical axis represents the count of number of voxels, *P*(*k*), as a function of the degree *k* of connectivity in the horizontal axis. Similar plots were obtained for the rest of the subjects.

**Fig 5:**
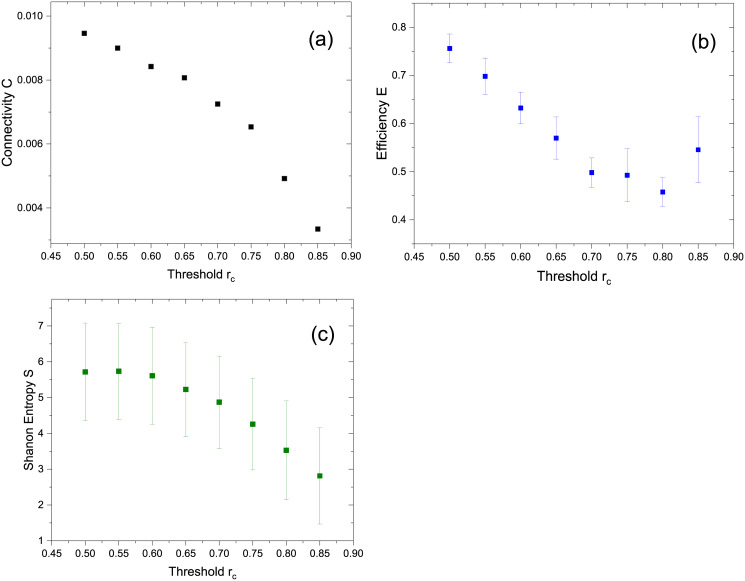
Clustering coefficient *C* for subject 1 (a) and averaged values of Efficiency *E* (b) and Shannon entropy *S* (c) for all the subjects as a function of the threshold *r*_*c*_.

As in the previous case, once the network is created after applying the paradigm-voxel filtering and the subsequent voxel-voxel correlation, the main topological graph measures were computed: *C*, *S*, *L* and *E* for all subjects. Fig 5(a) shows the clustering coefficient *C* for the same subject (subject 1) than Fig 4(a) for comparison where rest of subjects shows similar clustering coefficient behaviour as a function of the threshold. Average values of *E* and *S* for all subjects as a function of the threshold *r*_*c*_ from 0.50 up to 0.85 are shown in Figs 5(b) and 3(c). Log-log degree distributions could not be fitted for low value thresholds (from *r*_*c*_ = 0.50 up to 0.80) due to the non-linear behaviour of the log-log degree distribution plot. For all the subjects the averaged value of *γ* could be calculated for the highest value of *r*_*c*_ = 0.85 with a value *γ* = −0.83 ± 0.13.

### Voxel correlation in frequency domain

To avoid artifacts and any influence due to time shifting when correlating temporal voxel-voxel signals, the study was repeated in the frequency domain. Using the Fourier transform (FT) with real and imaginary components, as well as the power spectrum, all relevant measures were computed for all the subjects. Namely, the degree distribution fitting parameter *γ*, the clustering coefficient *C*, the Shannon entropy *S*, the characteristic path *L* and the network efficiency *E*. The results obtained were similar to those in the time domain.

### Graph parameters comparison

In a similar manner than Eguíluz et al., our present work include voxel-voxel correlation matrices of BOLD signals which were constructed from fMRI studies under right-handed finger tapping tasks for all subjects. As shown is Fig 2, a free-scale network was identified, where we considered a range of threshold values up to *r*_*c*_ = 0.90. However, the exponent of the degree distribution shows a linear dependence with threshold ranging from *γ* = −0.74 to *γ* = −1.79 as shown in averaged *γ* values in Fig 3(a) and 3(b) shows the dependence of the clustering coefficient with the threshold for subject 1. Similar results are obtained for the rest of the subjects. The average Efficiency *E* shows a minimum for an intermediate value of *r*_*c*_. As to the average Shanon entropy *S*, it shows a linear decrease with *r*_*c*_ in accordance to the degree distribution showing less dispersion as the threshold increases.

When the graphs are constructed with a prior filtering by correlation with the task paradigm the linear dependence of the distribution is lost for lower values of *r*_*c*_. As the value of the threshold increases the linear dependence seems to be recovered as shown in Fig 4 for the same subject 1 shown in Fig 2. For lower values of the threshold the distribution is similar to that of a random network with the average of the distribution located at the mean degree value. This results is consistent for all the subjects. The change of form in the distributions seems to appears close to the value of the threshold where *E* has a minimum.

All the network characterization parameters namely *C*, *E* and *S* behave in a similar manner as in the non-filtered case as can be oberved in Fig 5(a), 5(b) and 5(c). The log-log linear fitting of the degree distributions could only be achieve for those obtained with the highest values of the threshold with an average value of *γ* = −0.83 ± 0.13 for *r*_*c*_ = 0.85.

## Conclusion

Our findings show marked dependence of *γ* with the threshold, ranging from *γ* = −0.74 to *γ* = −1.79. As far as we know this behaviour has not been described before. Our results also suggest that for higher thresholds the free-scale behaviour of the graph is not related to the executed paradigm.

On the other hand the distributions of the graph at lower threshold, after eliminating the effects of paradigm execution, have random-like behaviour. This is the first study describing differences after filtering by paradigm execution. We suggest that paradigm signal prefiltering can provide more infomation about the brain networks when a task is involved. This particular behaviour in brain networks associated to volunteer-task studies (FTT-fMRI) shows new features not published before.

Further studies are in progress checking the possible applications of functional brain networks to characterize subjects with pathology since we believe that more information can be extracted from the functional brain networks where “there is plenty of room at the bottom”.

## Supporting information

S1 Appendix (PDF)

S2 Appendix (PDF)

S3 Appendix (PDF)

S4 Appendix (PDF)

S1 Data (CSV)

S2 Data (CSV)

S3 Data (CSV)
