# Supplementary material for: Prefiltering based on experimental paradigm for analysis of fMRI complex brain networks
Source: PLoS One. 2020 Oct 14;15(10):e0238994. doi: 10.1371/journal.pone.0238994 (PMC7556450; doi:10.1371/journal.pone.0238994)
Supplement: S1 Appendix — (PDF) [file pone.0238994.s001.pdf]

Los MADROÑOS
HOSPITAL

INFORMACIÓN Y AUTORIZACIÓN PARA PACIENTES. ESTUDIO DE RESONANCIA MAGNÉTICA.

Nombre y apellidos:__________________________________, citado el día:_______________a las:_________hs.

La Resonancia Magnética es una técnica de imágenes no-invasiva. Esta no emplea rayos-X, sino que obtiene las imágines mediante un campo magnético (estático) y ondas de radio-frecuencia.

Además, **usted debe saber** que:

1.- El estudio que se le realizará podrá durar hasta una hora, dependiendo de la complejidad del estudio

2.- La calidad final es muy dependiente de los movimientos, por lo que es muy importante que permanezca inmóvil durante todo el tiempo que dure la exploración.

3.- Durante la prueba oirá ruidos de diferentes intensidades. Estos son inevitables y necesarios, pero usted dispondrá de auriculares para mitigarlos.

4.- Estará comunicado en todo momento con el operador del aparato y dispondrá de un sistema de aviso instantáneo para cualquier imprevisto.

5.- Algunas personas podrían necesitar sedación o anestesia.

6.- En ocasiones es necesario el uso de contraste intravenoso basados en Gaolinio, cuya administración se decidirá por el médico responsable de la prueba durante la misma. Los posibles efectos secundarios del mismo son excepcionales.

7.- No debe utilizar maquillajes, sombras de ojos, rimmel, laca, etc., ya que pueden afectar notablemente la calidad de las imágenes.

8.- No se debe de entrar a la sala de exploración con ningún objeto metálico (pendientes, medallas, cadenas, orquillas, imperdibles, anillos, cinturón, etc.) teléfonos, relojes, gafas, lentillas audífonos, prótesis dentales

9.- Las tarjetas con bandas magnéticas (crédito, etc.) abonos transportes, cartillas de ahorro, etc., Pueden ser borradas si se introducen en la sala de exploración.

10.- En general le aconsejamos que no acuda a la realización del estudio con objetos que no vaya a necesitar.

Por favor, antes de someterse a la prueba, es importante que advierta al personal sanitario, en caso de ser portador de alguno de los siguientes objetos (marque con una cruz si posee alguno)

Marcapasos		Neuroestimulador		Bala, pedigón, metralla	
Grapas para aneurismas		Bomba de infusión		Esquirla metálica, ocular u orbitaria	
Grapas Quirúrgicas		Prótesis metálicas cardiacas		Dispositivo intrauterino	
Válvulas de derivación		Prótesis ortopédicas		Estimulador de crecimiento óseo	
Suturas metálicas		Prótesis de oídos		Diafragma	
Filtros vasculares		Prótesis del globo ocular		Tatuajes	
Alambres de embolización		Prótesis dental		Piercing	
Catéteres		Otras prótesis (vascular, biliar, etc.)			

Los MADROÑOS
HOSPITAL

En caso de tener algún tipo de prótesis, le recomendamos que acuda con la documentación técnica que tenga disponible. Muchas de ellas están hechas con materiales compatibles con esta técnica

Preparación: Puede tomar la medicación si la tiene prescrita. Puede hacer una vida totalmente normal antes y después de la exploración. Es conveniente que aporte toda la información clínica así como otras pruebas previas de las que disponga (Ecografías, TAC, radiografías, etc.).

Si usted tiene problemas de claustrofobias (miedo a los espacios cerrados) o si su peso supera los 120 Kg., háganoslo saber. Para cualquier pregunta o aclaraciones adicionales, por favor, no dude en consultar con el personal sanitario.

A continuación, por favor, rellene los siguientes campos:

De ser mujer: ¿Está usted embarazada? SI ☐ NO ☐

¿Ha sido sometido a alguna operación quirúrgica? SI ☐ NO ☐

En caso afirmativo, ¿de qué tipo?______________________________

____

Una vez leído este documento y cumplimentado el formulario, Don/Doña:________________

En nombre propio ☐ o como familiar responsable ☐, consiente que se le practique la exploración mediante Resonancia Magnética, estando informado de los riesgos de la misma.

Igualmente, SI ☐ NO ☐ consiente la administración de contrastes paramagnéticos si fuese necesario, conociendo los riesgos del mismo.

Consentimiento: Para que así conste, se firma la presente de conformidad en Madrid a ______de ____ ____________________________ de 201____.

DNI (Pasaporte, NIE, etc.)________________________ Firmado:____________________

Denegación: Para que así conste, se firma la presente de conformidad en Madrid a ______de ____ ____________________________ de 201____.

DNI (Pasaporte, NIE, etc.)________________________ Firmado:____________________
