# Supplementary material for: Prefiltering based on experimental paradigm for analysis of fMRI complex brain networks
Source: PLoS One. 2020 Oct 14;15(10):e0238994. doi: 10.1371/journal.pone.0238994 (PMC7556450; doi:10.1371/journal.pone.0238994)
Supplement: S2 Appendix — (PDF) [file pone.0238994.s002.pdf]

HOSPITAL
Los Madroños

Etiqueta

INFORMATION AND AUTHORIZATION FOR PATIENTS. MAGNETIC RESONANCE STUDY.

Name and surname: ____,
Date of Birth: ________________ Approximate Weight: ______
Dr. / Dra. prescribed by the test: ____
Reasonfor the Test: ____
Are you operated from the area to be explored? What operation did they perform?____
Known allergies: ____

Magnetic Resonance is a non-invasive imaging technique. It does not use X-rays, but obtains the images by means of a magnetic field (static) and radio-frequency waves.

Also, you should know that:

1.- The study that will be carried out may last up to one hour, depending on the complexity of the study. The final quality is highly dependent on movements, so it is very important that you remain motionless for the entire duration of the examination.
2.- During the test you will hear noises of different intensities. These are unavoidable and necessary, but you will have headphones / earplugs to mitigate them.
3.- You will be in communication at all times with the operator of the device and will have an instant warning system for any unforeseen event.
4.- Sometimes it is necessary to use intravenous contrast based on Gaolinium, whose administration will be decided by the doctor responsible for the test during the test. The possible side effects of it are exceptional.
5.- Do not enter the examination room with any metallic object (earrings, medals, chains, hairpins, safety pins, rings, belt, etc.), telephones, watches, glasses, hearing aid lenses, dental prostheses. Cards with magnetic stripes (credit, etc.), transport passes, savings books, etc.,
They can be erased if they enter the examination room.
6.- In general we advise you not to go to the study with objects that you will not need.
Please, before undergoing the test, it is important that you warn the health personnel if you are carrying any of the following objects (mark with a cross if you have any)

Pacemaker		Neurostimulator		Bullet, pedigree, shrapnel	
Aneurysm Staples		Infusion Pump		Metal, Eye, Or Orbital Shard	
Surgical Staples		Staples Metal heart prostheses		Intrauterine device	
Bypass valves		Orthopedic prostheses		Bone growth stimulator	
Metal sutures		Ear prosthesi		Diaphragm	
Vascular filters		Eyeball prosthesis			
Embolization wires		Dental prosthesis		Piercing	
Catheters		prostheses (vascular, biliary, etc.)			

HOSPITAL
Los Madroños

Etiqueta

In case of having any type of prosthesis, we recommend that you go with the technical documentation that you have available. Many of them are made with materials compatible with this technique

Preparation: You can take the medication if you have it prescribed. You can lead a totally normal life before and after the scan. It is convenient that you provide all the clinical information as well as other previous tests that you have (ultrasound, CT, x-rays, etc.).

If you have problems with claustrophobia (fear of closed spaces) or if your weight exceeds 120 kg, let us know. For any further questions or clarifications, please do not hesitate to ask the healthcare staff.

Then please fill in the following fields:

If you are a woman: Are you pregnant? YES □ NO □

Are you undergone any surgical operation? YES □ NO □

If yes, what type?

After reading this document and filling in the form, Mr. / Mrs.:
On your own behalf □ or as a responsible family member □, you consent to have the MRI scan performed, being informed of its risks.

Likewise, YES □ NO □ consent to the administration of paramagnetic contrasts if necessary, knowing the risks of the same.

CONSENT: For the record, this agreement is signed in Madrid on_____to ________________________ of 202___.

X

Identity Number (Passport, NIE, etc.)______________________________ Signature:______________________

Refusal: For the record, this agreement is signed in Madrid on____ of 202____.

Identity Number (Passport, NIE, etc.)____________________________________ Signature:________________________________
