# Supplementary material for: Prefiltering based on experimental paradigm for analysis of fMRI complex brain networks
Source: PLoS One. 2020 Oct 14;15(10):e0238994. doi: 10.1371/journal.pone.0238994 (PMC7556450; doi:10.1371/journal.pone.0238994)
Supplement: S3 Appendix — (PDF) [file pone.0238994.s003.pdf]

CONSENTIMIENTO PARA PARTICIPAR EN ESTUDIO DE INVESTIGACIÓN DE NEUROIMAGEN MEDIANTE RESONANCIA MAGNÉTICA

Título del Proyecto: Tractografía Funcional de Imagen de Resonancia Magnética.

Investigador Principal: Dr. Alberto Muñoz (Dpto. de Radiología, Facultad de Medicina, Universidad Complutense de Madrid)

Co-investigadores: Dr. Leoncio Garrido (Instituto de Ciencia y Tecnología de Polímeros, Madrid) y Jesús Tornero (Hospital Los Madroños, Brunete, Madrid)

Por favor, preste atención a la siguiente información antes de decidirse a participar en el estudio.

Propósito de la investigación:

El objetivo principal es investigar y cuantificar los impulsos eléctricos que transmiten haces de fibras nerviosas importantes del cerebro (en particular, el haz corticoespinal) al realizar un movimiento voluntario de una extremidad (por ejemplo, una mano o brazo).

Consultas sobre el estudio:

Si tiene alguna pregunta sobre el estudio, puede ponerse en contacto con los investigadores responsables del estudio, los Drs. Alberto Muñoz (610399188), Leoncio Garrido (91 561 8808, ext.319) y Jesús Tornero (650036129).

¿Qué hará Ud. durante el procedimiento?

Su participación consiste en realizarse una prueba de resonancia magnética de tiempo breve, aproximadamente 50 minutos, durante la cual Ud. permanecerá tumbado en la mesa-camilla del imán, como en una exploración clínica normal, y se le solicitará que abra y cierre su mano en una cadencia que lo escuchará por un altavoz, y alternativamente movilizará la mano o la dejará quieta.

Es importante que permanezca inmóvil durante la exploración para que la prueba no salga "errónea" y mantenga los ojos cerrados.

Durante el procedimiento oirá diferente tipos de sonidos provenientes del imán, parecidos a un taconeo o llamadas a una puerta, debido a los cambios en la intensidad de los campos magnéticos. Por si fueran desagradables se le dotará de tapones acústicos para disminuir la sensación de ruido.

Si durante el procedimiento se siente incómodo y desea detener el estudio o abandonar la sala, simplemente dígalo o apriete un dispositivo que se le indicará y la exploración quedará finalizada inmediatamente, así como su desalojo de la sala.

El resultado del estudio se comparará con otros similares de otros voluntarios. Sus resultados permanecerán anónimos y solo se identificarán mediante un código.

En ocasiones se le podría solicitar repetir el estudio o ampliarlo para obtener información adicional. Si fuera así, su participación sería de nuevo voluntaria y opcional.

El estudio no tiene un propósito o finalidad de valoración de su cerebro desde la perspectiva clínica o neurológica. Por ello no recibirá un informe médico del estudio.

Si se detectara una anomalía significativa, los investigadores se pondrían en contacto con un neurólogo para considerar una consulta formal neurológica.

El estudio no conlleva la realización de ningún procedimiento invasivo, ni la inyección de contraste.

Duración del estudio:

La duración el examen de RM es de aproximadamente 50 minutos.

Riesgos:

Los estudios de RM utilizan altos campos magnéticos. No se conocen ni se piensa que puedan existir riesgos de ningún tipo sobre la salud en humanos, tal y como se hacen estudios de Resonancia Magnética clínica (los indicados por los médicos como pruebas de imagen).

No obstante, en estos estudios no pueden participar sujetos con prótesis que se activen de forma eléctrica, magnética o mecánica, o con grapas vasculares, o con otras prótesis o cuerpos extraños metálicos ferromagnéticos o marcapasos.

Si el participante es mujer y cree o podría estar embarazada, se le recomienda no realizar el estudio.

Asimismo si Ud. tiene o cree tener claustrofobia se le recomienda no realizar el estudio.

Beneficios:

Teniendo en cuenta que en el estudio se emplea un nuevo método de imagen de RM, no obtendrá ningún beneficio, aunque, si el estudio demuestra que el método es útil, es posible que los resultados obtenidos puedan ser relevantes para el diseño de métodos de IRM basados en difusión que mejoren el diagnóstico y seguimiento de patologías y terapias que afecten al sistema nervioso central. En cualquier caso, al finalizar el estudio de todos los participantes se le explicará detalladamente las conclusiones obtenidas.

Los resultados de la investigación podrán ser publicados en libros o revistas científicas o utilizados en enseñanza. No obstante, en ningún caso aparecerá su nombre u otros posibles elementos identificativos sin su permiso expreso y firmado. En caso de ser publicado podrá solicitar una copia de la investigación.

Compensaciones:

No recibirá gratificación económica.

Los investigadores participantes no reciben gratificación económica por la realización de este estudio y declaran no tener conflicto de intereses que puedan condicionar los resultados de este estudio.

Confidencialidad:

Su participación en el estudio es confidencial y su identidad será protegida en todo momento. Así, la información obtenida será guardada por los investigadores responsables del estudio e identificada únicamente con un código alfanumérico. El registro que establece la correspondencia entre el nombre del sujeto y la información correspondiente se guardará en un lugar seguro y separado del resto.

Acceso, cancelación, rectificación y oposición al estudio:

Su participación en el estudio es completamente voluntaria. No está en la obligación de participar. Si decide hacerlo, puede cambiar de opinión en cualquier momento y solicitar la exclusión del estudio a cualquiera de los investigadores responsables de la investigación. Su salida del estudio no afectará en ningún aspecto su atención y cuidados médicos presentes o futuros en el Hospital. Asimismo, el investigador principal de la investigación puede terminar su participación en el estudio en cualquier momento, después de que haya explicado los motivos que han conducido a ello.

Consentimiento:

La naturaleza y propósito del estudio me ha sido suficientemente explicada y estoy de acuerdo a participar en el estudio.

También conozco que puedo abandonar el estudio en cualquier momento.

Firma : ____ Fecha: ___________________

Nombre: ____
