# Supplementary material for: Prefiltering based on experimental paradigm for analysis of fMRI complex brain networks
Source: PLoS One. 2020 Oct 14;15(10):e0238994. doi: 10.1371/journal.pone.0238994 (PMC7556450; doi:10.1371/journal.pone.0238994)
Supplement: S4 Appendix — (PDF) [file pone.0238994.s004.pdf]

CONSENT TO PARTICIPATE IN NEUROIMAGING RESEARCH STUDY THROUGH MAGNETIC RESONANCE

Project Title: Magnetic Resonance Imaging Functional Tractography.

Principal Investigator: Dr. Alberto Muñoz (Department of Radiology, Faculty of Medicine, Complutense University of Madrid)

Co-researchers: Dr. Leoncio Garrido (Institute of Polymer Science and Technology, Madrid) and Dr. Jesús Tornero (Hospital Los Madroños, Brunete, Madrid)

Please pay attention to the following information before deciding to participate in the study.

Research purpose:

The main objective is to investigate and quantify the electrical impulses that transmit important nerve fiber bundles from the brain (in particular, the corticospinal bundle) when performing a voluntary movement of a limb (for example, a hand or arm).

Questions about the study:

If you have any questions about the study, you can contact the researchers responsible for the study, Dr. Alberto Muñoz (610399188), Dr. Leoncio Garrido (91 561 8808, ext. 319) and Dr. Jesús Tornero (650036129).

What will you do during the procedure?

Your participation consists of undergoing a short-time magnetic resonance test, approximately 50 minutes, during which you will lie down on the magnet table-stretcher, as in a normal clinical examination, and you will be asked to open and close your hand in a cadence that you will hear through a loudspeaker, and alternately mobilize your hand or hold it still.

It is important that you remain still during the scan so that the test does not come out “wrong” and that you keep your eyes closed.

During the procedure, you will hear different types of sounds coming from the

magnet, similar to clicking or knocking on a door, due to changes in the strength of the magnetic fields. In case they were unpleasant, it will be fitted with acoustic plugs to reduce the sensation of noise.

If you feel uncomfortable during the procedure and wish to stop the study or leave the room, simply say so or squeeze a device that will be prompted and the scan will be immediately completed and your removal from the room.

The result of the study will be compared with similar results from other volunteers. Your results will remain anonymous and will only be identified by a code.

Sometimes you may be asked to repeat the study or expand it to obtain additional information. If so, your participation would again be voluntary and optional.

The study is not intended to evaluate your brain from a clinical or neurological perspective. Therefore, you will not receive a medical report from the study.

If a significant abnormality was detected, the researchers would contact a neurologist to consider a formal neurological consultation.

The study does not involve the performance of any invasive procedure, or the injection of contrast.

Study duration:

The duration of the MRI exam is approximately 50 minutes.

Risks:

MRI studies use high magnetic fields. It is not known or thought that there may be risks of any kind on human health, as clinical Magnetic Resonance studies are done (those indicated by doctors as imaging tests).

However, these studies cannot include subjects with prostheses that are activated electrically, magnetically, or mechanically, or with vascular staples, or with other prostheses or ferromagnetic metallic foreign bodies or pacemakers.

If the participant is a woman and believes or could be pregnant, it is recommended not to conduct the study.

Likewise, if you have or think you have claustrophobia, it is recommended that you not carry out the study.

Benefits:

Taking into account that the study uses a new MRI imaging method, it will not obtain any benefit, although, if the study shows that the method is useful, it is possible that the results obtained may be relevant for the design of MRI methods. based on diffusion that improve the diagnosis and monitoring of pathologies and therapies that affect the central nervous system. In any case, at the end of the study of all participants, the conclusions obtained will be explained in detail.

The results of the research may be published in books or scientific journals or used in teaching. However, in no case will your name or other possible identifying elements appear without your express and signed permission. If it is published, you may request a copy of the research.

Compensations:

You will not receive financial gratification.

Participating researchers do not receive financial rewards for conducting this study and declare that they have no conflicts of interest that may condition the results of this study.

Confidentiality:

Your participation in the study is confidential and your identity will be protected at all times. Thus, the information obtained will be saved by the researchers responsible for the study and identified only with an alphanumeric code. The record that establishes the correspondence between the subject's name and the corresponding information will be kept in a safe place and separate from the rest.

Access, cancellation, rectification and opposition to the study:

Your participation in the study is completely voluntary. You are not required to participate. If you decide to do so, you can change your mind at any time and ask any of the researchers responsible for the research to be excluded from the study. Your withdrawal from the study will not affect in any way your present or future

medical care and attention at the Hospital. Also, the principal investigator of the research may terminate her participation in the study at any time, after she has explained the reasons that led to it.

Consent:

The nature and purpose of the study has been sufficiently explained to me and I agree to participate in the study.

I also know that I can leave the studio at any time.

Signature: ___________________________________Date: ___________________

Name: ____
